# Case Report: Non-negligible Epstein-Barr virus-associated posttransplant lymphoproliferative disorders in a lung transplant recipient

**DOI:** 10.3389/fimmu.2023.1244534

**Published:** 2023-09-15

**Authors:** Juan Hu, Yong-Wei Yu, Dong-Sheng Han, Xue-Jie Li, Yi-Qi Zhang, Hong-Liu Cai, Yong-Hong Xiao, Xia Zheng

**Affiliations:** ^1^ Intensive Care Unit, The First Affiliated Hospital, Zhejiang University School of Medicine, Hangzhou, China; ^2^ Centre of Clinical Laboratory, The First Affiliated Hospital, Zhejiang University School of Medicine, Hangzhou, China; ^3^ Department of Pathology, The First Affiliated Hospital, Zhejiang University School of Medicine, Hangzhou, China; ^4^ State Key Laboratory for Diagnosis and Treatment of Infectious Diseases, National Clinical Research Center for Infectious Diseases, Collaborative Innovation Center for Diagnosis and Treatment of Infectious Diseases, The First Affiliated Hospital, Zhejiang University School of Medicine, Hangzhou, China

**Keywords:** Epstein-Barr virus, posttransplant lymphoproliferative disorders, lung transplant, immunosuppression, invasive fungal disease

## Abstract

**Background:**

Posttransplant lymphoproliferative disorders (PTLDs) are uncommon but serious complications in patients following solid organ transplantation. Primary Epstein-Barr virus (EBV) infection is a risk factor for the development of PTLD, especially early-onset PTLD, in EBV-negative recipients. To date, however, there are no specific guidelines on the threshold of EBV-DNA load for therapeutic intervention, the source for measurement (e.g., blood, bronchoalveolar fluid), or the use of antiviral agents as prophylaxis for early PTLD prevention in EBV-mismatched patients.

**Methods:**

The present study describes a 56-year-old male lung transplant recipient diagnosed with EBV-associated PTLD.

**Results:**

This patient had a history of invasive fungal disease and Mucor and Aspergillus fumigatus infections in the early post-transplant period, necessitating antifungal therapy throughout the course of the disease. The patient was EBV-positive 15 days after transplantation, with lung CT showing multiple bilateral nodules of varying sizes beginning 98 days after transplantation. A lung biopsy showed PTLD, and next-generation sequencing (NGS) revealed EBV. This patient, however, did not receive any antiviral therapy for early PTLD prevention or any PTLD-related treatment. He died 204 days after lung transplantation.

**Conclusion:**

The present study describes a lung transplant recipient who developed EBV-associated PTLD, a non-negligible disease, after solid organ transplantation. Monitoring EBV-DNA load is important, as a sudden increase may be a sensitive indicator of PTLD. An earlier diagnosis may increase the likelihood of successful treatment.

## Introduction

Posttransplant lymphoproliferative disorders (PTLDs) are the second most common group of malignancies after skin cancer, occurring after solid organ transplantation (1–4). PTLDs are regarded as a devastating complication of organ transplantation (5–7), with mortality rates ranging from 50% to 70% (8–12). Lung transplant recipients have a higher overall incidence of PTLD (3%–9%) (7, 13, 14) than renal (1.4%–2.9%) and liver (0.9%–2.6%) transplant recipients (15, 16). For all of these patients, the risk of PTLD is highest during the first year after transplantation, with differences during this period being largely responsible for the overall differences among recipients of these organs (17).

The clinical and morphologic presentations of PTLD, including its histopathologic features and organ involvement, vary greatly (18). These symptoms can range from benign lymphoproliferation and infectious mononucleosis-like features to invasive neoplastic processes such as classical Hodgkin lymphoma. The predominant risk factors include primary Epstein-Barr virus (EBV) infection and the level of immunosuppression following transplantation (18, 19). EBV-naive patients who receive an organ from an EBV-infected donor are at highest risk of developing PTLD (20). PTLD is definitively diagnosed by pathologic examination (19), and the presence of EBV in a tumor is based on EBV-encoded RNA (EBER) *in situ* hybridization (21). Treatment is dependent on the type of organ transplanted and the location and extent of the disease, with reduction of immunosuppression being the most important management modality. Because patients with PTLD have a poor prognosis, early detection and diagnosis are critical.

The present study describes a 56-year-old male lung transplant recipient who developed EBV-associated PTLD and died within 3 days of diagnosis. This report emphasizes the importance of monitoring EBV-DNA in organ transplant recipients who are at high risk of PTLD and of including PTLD in the differential diagnosis of transplant recipients who present postoperatively with a lung mass and EBV-DNA positivity, even in patients with a history of invasive pulmonary aspergillosis.

## Case description

A 56-year-old male patient, who had been unable to perform daily activities for 2 years, was admitted to our hospital for a lung transplant. He had developed pulmonary tuberculosis during his college years, followed by a co-infection with invasive pulmonary aspergillosis. His condition worsened, and he could only walk 10 meters indoors under nasal cannula oxygenation. He became increasingly emaciated (body mass index 15.6 kg/m^2^), and he was repeatedly hospitalized for pneumothorax. Pulmonary function microscopy revealed extremely restrictive ventilation and severely reduced carbon monoxide diffusion. A lung transplant had been recommended 2 years earlier. A preoperative examination showed that he was negative on a T-spot test, on a smear test for Mycobacterium tuberculosis in sputum, and on a culture of sputum and blood, but positive on a galactomannan (GM) test. Next-generation sequencing (NGS) of sputum samples showed that he was positive for Aspergillus fumigatus but negative for M. tuberculosis. Preoperative blood tests showed that he was negative for EBV-DNA, cytomegalovirus (CMV)-DNA, and EBV-IgM and CMV-IgM, but positive for EBV-IgG and CMV-IgG. Cranial and abdominal computed tomography (CT) scans and superficial lymph node ultrasound showed normal results, and the patient’s serum was negative for tumor markers.

The lung donor was a 30-year-old man who had experienced a cerebral hemorrhage. He was negative for blood and sputum cultures, a M. tuberculosis sputum smear, and CMV-DNA and EBV-DNA tests, but was not tested for antibodies to EBV and CMV. Preoperative imaging examinations, including chest and abdominal CT and superficial lymph node ultrasound, showed no abnormalities, and his serum was negative for tumor markers. Human leukocyte antigen (HLA) matching data between donor and recipient were not available.

**Figure 1:**
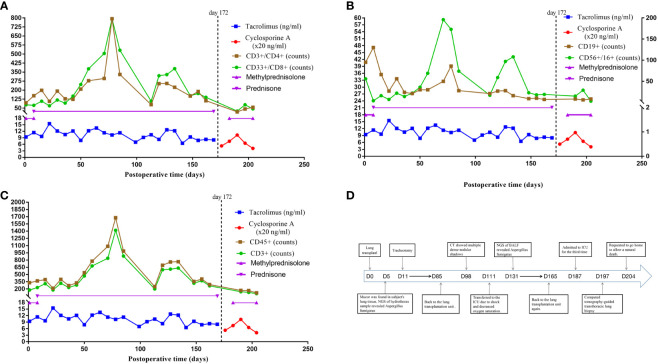
Immunosuppressive therapy and immune cell counts after lung transplant and a timeline depicting the disease course of the patient. **(A)** The immunosuppressive therapy of the case after lung transplant and the trends of immune cell counts of CD3+/CD4+ and CD33+/CD8+. **(B)** The immunosuppressive therapy of the case after lung transplant and the trends of immune cell counts of CD19+ and CD56+/CD16+. **(C)** The immunosuppressive therapy of the case after lung transplant and the trends of immune cell counts of CD45+ and CD3+. **(D)** The timeline illustrates the different events in the course of the patient’s treatment and disease progression. CD3+ T cells (reference range 797-2370), CD3+/CD4+ helper T cells (reference range 432-1341), CD3+/CD8+ cytotoxic T cells (reference range 238-1075), CD19+ B cells (reference range 86-594), CD56+/CD16+ NK cells (reference range 127-987), CD45+ absolute lymphocyte count (reference range 800-594).

The patient underwent a double lung transplant on 15/05/2022. Severe chest adhesions prolonged lung transplantation by more than 12 hours, and the recipient experienced a right phrenic nerve injury. Postoperatively, the recipient was treated with conventional immunosuppressive agents such as tacrolimus (target trough concentration 10–12 ng/mL) and prednisolone (0.5 mg/kg/d for 1 week, followed by a gradual reduction to 0.25 mg/kg/d) (**Figures 1A–C**). He also received antifungal treatment with intravenous voriconazole and caspofungin plus nebulized amphotericin B, in addition to isoniazid (300 mg/day for 3 months) to prevent M. tuberculosis infection. Because he experienced an acute kidney injury on the first postoperative day, however, he was not administered prophylactic antiviral therapy.

Five days after transplantation, Mucor was detected in the lung tissue of the recipient, with NGS of a hydrothorax sample showing the presence of A. fumigatus. Voriconazole and caspofungin were discontinued, and the patient was started on intravenous posaconazole injections for antifungal treatment. A tracheotomy was performed 11 days after transplantation, and rehabilitation exercises were gradually introduced. Following this, there was no evidence of Aspergillus fumigatus. Beginning 45 days after transplantation, the subject required only oral isavuconazole capsules as an antifungal regimen. Monitoring of immune function indicators showed that his T-cell counts were below normal, with the numbers of helper and cytotoxic T-cells being 123 and 150 cells/ul, respectively (**Figures 1A–C**). He went back to the lung transplantation unit on postoperative day 85, receiving continuous mechanical ventilation, as shown in the timeline in **Figure 1D**.

**Figure 2:**
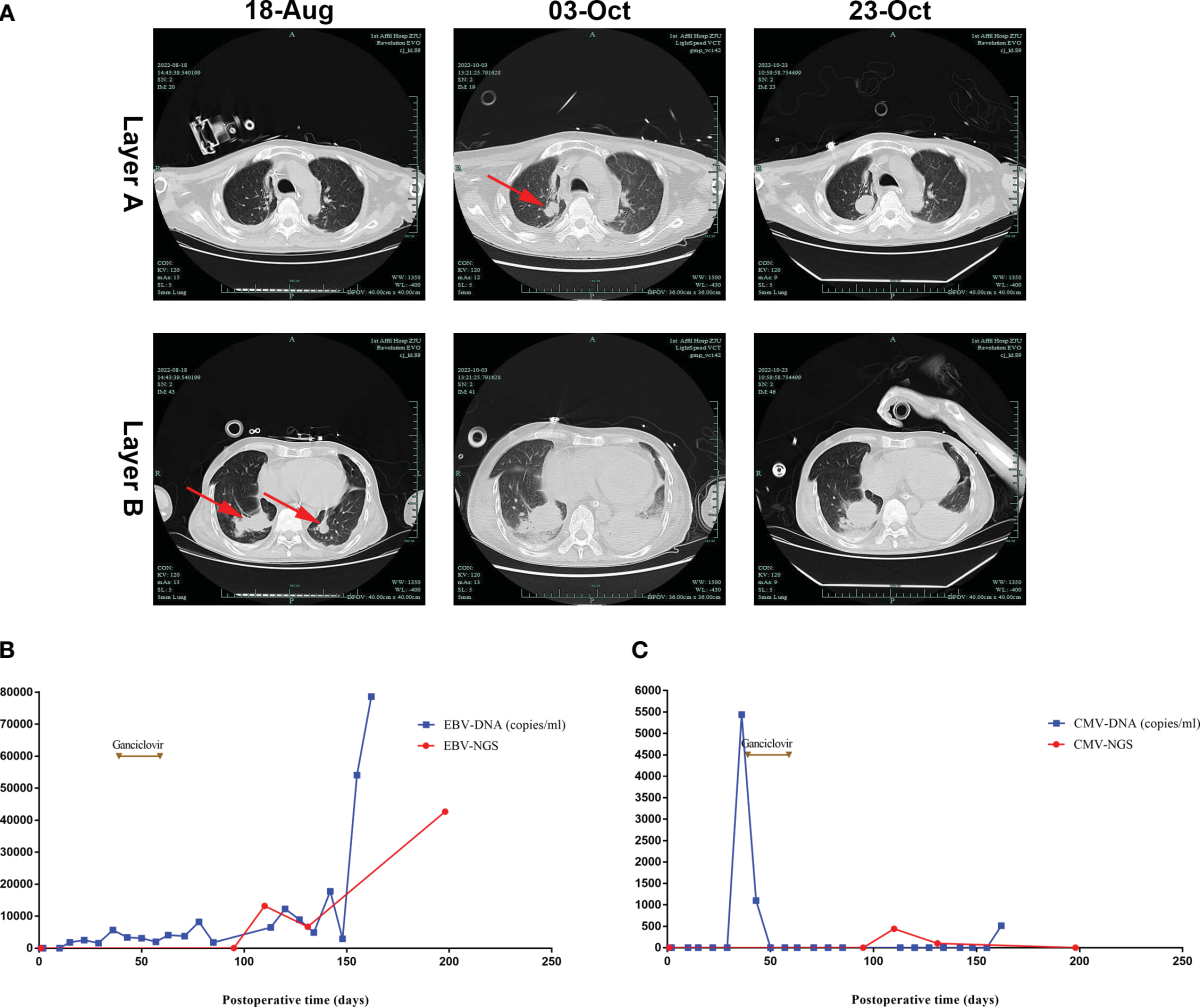
Lung CT and serum EBV/CMV monitoring after lung transplantation. **(A)** Lung CT scan showing that dense nodular shadows were seen in both lower lungs on August 18th, then larger and more nodular and patchy dense shadows were seen on October 3rd and 23rd. **(B)** EBV-DNA was negative at the beginning, then was detected in the blood on postoperative day 15, with an increase in viral load in the late stage. **(C)** CMV-DNA was negative at the beginning, then was positive on postoperative day 36.

However, the patient experienced chest tightness and shortness of breath, and his condition worsened. A lung CT scan on day 98 showed multiple dense nodular shadows in both lower lungs (**Figure 2A**). He had no fever, cough, or hemoptysis. Routine laboratory values, which included a complete blood count and procalcitonin (PCT) and C-reactive protein (CRP) concentrations, were within normal limits. He was negative for T-spot and tuberculosis smear tests, in addition to being negative for tumor markers. A sputum culture suggested infection with Pseudomonas aeruginosa. NGS showed that his bronchioalveolar lavage fluid (BALF) was positive for P. aeruginosa, Corynebacterium striatum, EBV, and CMV. Although there was no evidence of fungus, the possibility of invasive pulmonary aspergillosis was considered, and micafungin (250 mg qd) was added as another antifungal agent for the patient at once.

**Figure 3:**
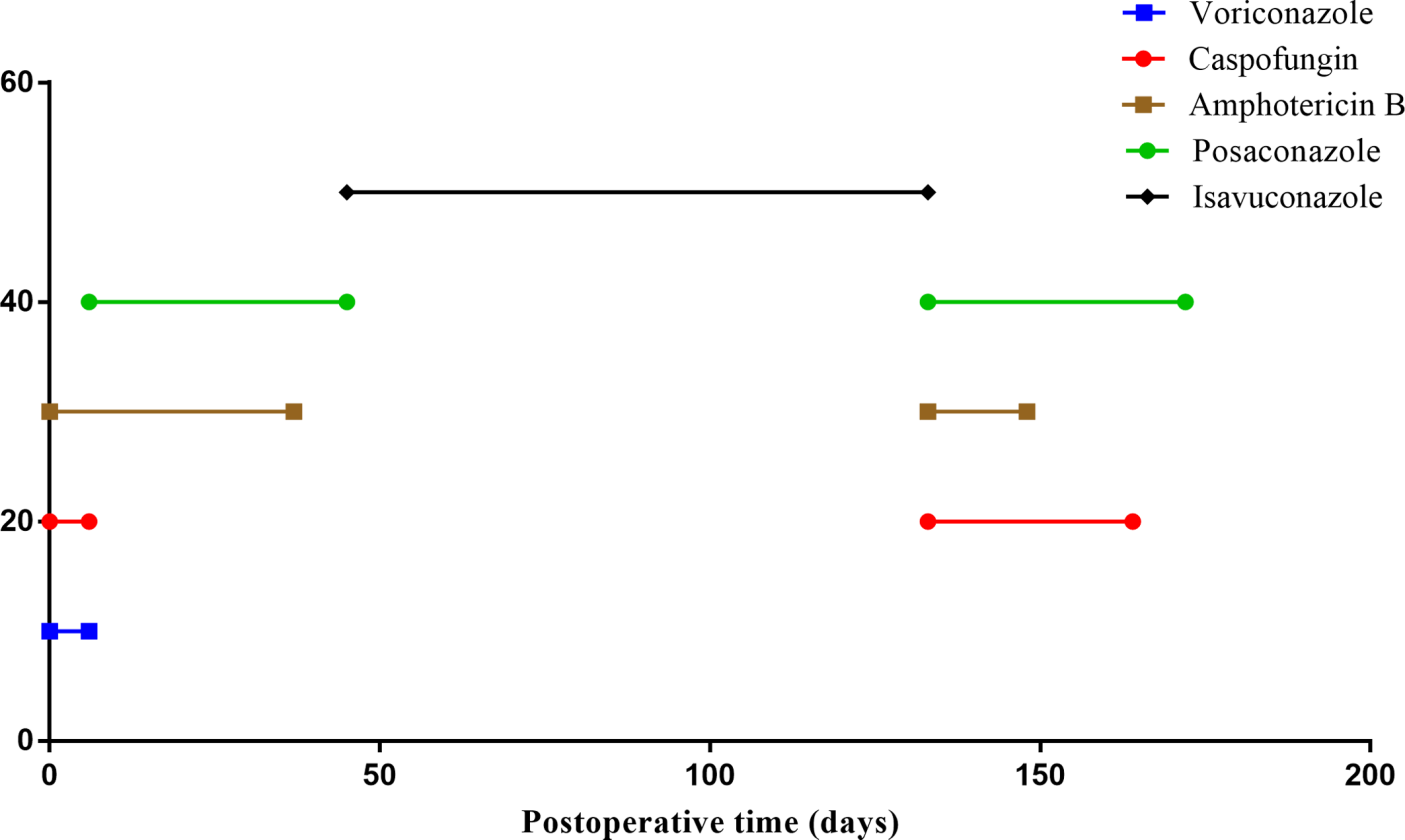
Antifungal therapy after lung transplant. All antifungal drugs received by the patient after lung transplant; the X-axis represents treatment time.

On postoperative day 111, the patient was transferred to the ICU due to shock and decreased oxygen saturation. A sputum culture revealed the presence of P. aeruginosa, with blood tests showing a CRP concentration of 3.55 mg/L, a PCT concentration of 2.62 ng/mL, a white blood cell count of 14.41*10^9^/L, a hemoglobin concentration of HB 50 g/L, and a platelet (PLT) count of 63*10^9^/L. The total lymphocyte count was extremely low (**Figures 1A–C**). NGS of BALF suggested the presence of A. fumigatus, EBV, and CMV, but bone marrow tests showed no tumor cells. Although his antifungal therapy was changed (**Figure 3**) based on the NGS results, the patient’s condition continued to deteriorate, and he was treated with continuous renal replacement therapy for acute kidney injury. Moreover, lung CT (**Figure 2A**) showed progressively larger and more nodular and patchy dense shadows in his lungs, but the patient and his family refused lung tissue biopsy.

**Figure 4:**
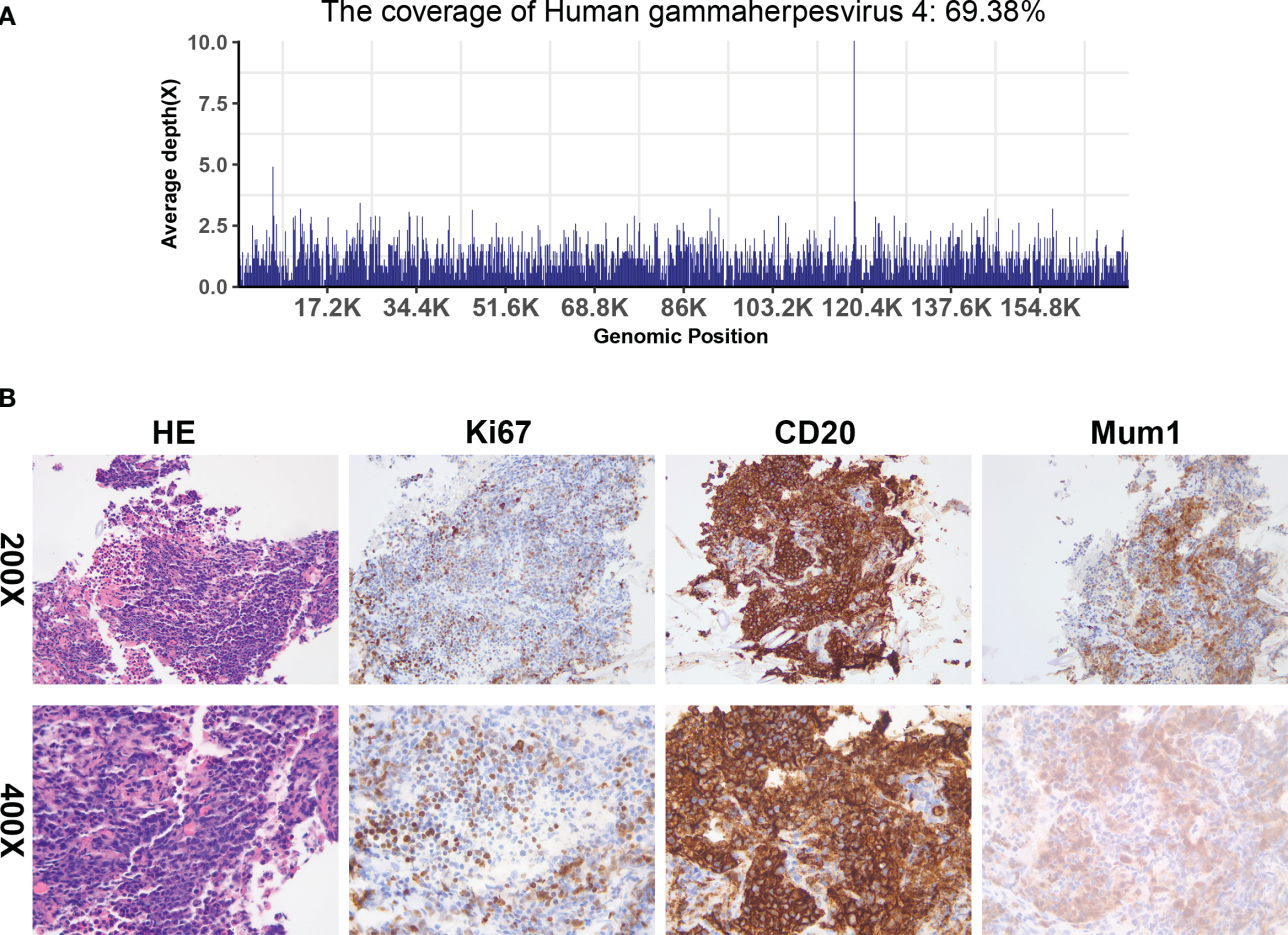
Next-generation sequencing and histologic evaluation of puncture samples from right lung masses. **(A)** mNGS result of nucleotide sequences distributed along the genome of EBV in the case (the X-axis represents the full-length reference genome and the Y-axis represents the distribution of detected sequences at different locations in the reference genome). **(B)** Tissue biopsy shows an aggregation of abnormal lymphocytes (HE x200 and x400), and an immunohistochemical examination of these cells revealed a positive reaction to CD20 (x200 and x400), Ki-67 (x200 and x400), and Mum1 (x200 and x400).

On postoperative day 187, the patient’s consciousness deteriorated. High-dose vasopressors were administered to maintain blood pressure, and high mechanical ventilation parameters were set to maintain oxygen saturation. A series of blood tests revealed that his condition had deteriorated further. Monitoring of immune function indicators showed a state of severe immunosuppression. The patient’s family consented to a lung tissue biopsy, and a CT-guided transthoracic lung biopsy was performed on postoperative day 197. NGS of the lung tissue sample revealed EBV (**Figure 4A**), and the tissue biopsy showed PTLD (B cell, monomorphic), with a tendency to develop diffuse large B cell lymphoma (**Figure 4B**). Immunohistochemical assays showed that the tissue samples were positive for CD45, RB1, BRG1, CD20, and MUM1; partially positive for CD19, Bcl-2, and Ki-67 (50%); and negative for CK (pan), P40, TTF-1, CA, Syn, CD56, P53, CD117, CD10, Bc-6, CD5, Bcl-2, and C-Myc. These findings suggested EBV-associated PTLD.

In reviewing the patient’s treatment, we downplayed the effects of EBV because the patient was positive for EBV-IgG and CMV-IgG before lung transplant, and he did not receive any antiviral drugs (ganciclovir or acyclovir) during the early posttransplantation phase. Viral loads of EBV and CMV were monitored weekly (**Figures 2B, C**), with the results suggesting that the patient was initially negative for EBV-DNA and CMV-DNA; his blood concentration of EBV-DNA was 1840 copies/ml on day 15 and his blood concentration of CMV-DNA was 5440 copies/mL on day 36, at which time antiviral therapy (ganciclovir 250 mg bid) was started. This treatment was discontinued on postoperative day 59 due to leukopenia. Although late viral monitoring (EBV-DNA) and NGS of BALF showed the persistence of EBV positivity with an increase in viral load during the late stage, the patient did not receive any antiviral therapy thereafter.

Three days after the transthoracic lung biopsy (i.e., on postoperative day 200), the patient experienced respiratory cardiac arrest; there was no choice but to execute positron emission tomography and computed tomography (PET-CT), and he did not receive any PTLD-related treatment. On postoperative day 204, the patient’s family requested that he be transferred home to die naturally.

## Discussion

This report describes a 56-year-old lung transplant recipient with a history of pulmonary tuberculosis and invasive pulmonary aspergillosis. On the fifth day after transplantation, Mucor was found in the recipient’s lung tissue, and NGS of a hydrothorax sample revealed A. fumigatus. We then paid so much attention to fungal infection that we neglected the possibility of PTLD when lung CT showed multiple dense nodular shadows in both lower lungs on postoperative day 98, although EBV-DNA monitoring was positive and there was no evidence of fungus at that time. A lung tissue biopsy showed PTLD (B cells, monomorphic), and lung tissue NGS showed EBV. However, his condition deteriorated rapidly, and there was no opportunity to treat his PTLD before he died on postoperative day 204.

PTLD is an uncommon but serious complication in solid organ transplant recipients and is the most frequent cause of cancer-related mortality in this population. The first reports of PTLD were published in 1968 (22), and the term PTLD was coined in 1984 (23). PTLDs can be subclassified into six types: plasmacytic, infectious mononucleosis-like, florid follicular hyperplastic, polymorphic, monomorphic, and classic Hodgkin lymphoma PTLDs (24). Monomorphic PTLD, present in 60%–80% of patients with PTLD, is the most common subtype, with ~90% of monomorphic PTLD being of B cell origin (25). The incidence of PTLD is dependent on the type of organ transplanted (17), being most frequent in intestinal transplant recipients (up to 32%), and lung transplant recipients being at moderate risk for the development of PTLD (13). The clinical symptoms of PTLD may be non-specific (fever, night sweats, weight loss, allograft dysfunction) or related to problems at the site of the lymphoid mass (lymph node enlargement or symptoms in the gastrointestinal tract, brain, liver, lung, or kidney) (13). Although pathologic testing remains definitive in the diagnosis of PTLD, radiographic imaging, e.g., CT, MRI, and PET-CT, can be used in the delineation, diagnosis, and staging of PTLD and to assess response to treatment (19).

Although the exact pathogenesis of PTLD remains unclear, primary EBV infection is a risk factor for the development of PTLD in EBV-negative recipients. Oncogenic EBV drives abnormal lymphocyte proliferation in 60%–80% of PTLDs (26), especially in early-onset disease, occurring <1–2 years after transplantation. The etiologic triggers in the remaining 20%–50% of EBV-negative PTLDs have not been determined (13). EBV enters the body through the laryngopharynx, penetrates the mucosal epithelium, and infects submucosal B cells, inducing the expression of viral genes as the infected cells pass through the lymph node germinal centers and mature. Some latently infected memory B cells leave the germinal centers and persist, while other infected memory cells evolve into plasma cells that shed newly assembled free virions into the saliva. The proliferation of B cell blasts in immunocompetent individuals is inhibited by EBV-specific CD8-expressing cytotoxic T cells (CTLs) (27). In immunocompromised solid organ transplant recipients, however, cytotoxic T-cell function is diminished, resulting in uncontrolled proliferation of EBV-infected B cells and eventual development of PTLD (13).

EBV mismatch (donor (D)+/recipient (R)-) is also an important risk factor for early EBV+PTLD (28–30). EBV-associated PTLD has been observed in 30–40% of EBV-seronegative recipients who receive EBV-seropositive donor lungs (31, 32), but in <5% of EBV-seropositive recipients who receive EBV-seropositive donor lungs (32). The risk of developing PTLD after transplantation is reported to be 200-fold higher in EBV-seronegative than in EBV-seropositive lung recipients (31). In the present case, both the donor and the recipient were negative for EBV-DNA before lung transplantation; although the recipient was positive for EBV-IgG, this was not tested in the donor, and as there was no information about HLA matching status, we could not make any conclusion on D/R EBV match or mismatch, which was different from the previous research (19, 33). The recipient was positive for EBV DNA (1840 copies/mL) 15 days after transplantation, with subsequent viral monitoring (EBV-DNA) and NGS of BALF showing that this patient remained EBV positive, with an increase in viral load and NGS showing the presence of EBV in a lung biopsy sample. These findings suggested that PTLD in this patient was associated with an EBV infection. However, because the donor was negative for EBV-DNA and serum tumor markers, superficial lymph node ultrasound, and chest CT showed no abnormalities, the EBV responsible for PTLD likely did not originate from the transplant donor.

CMV mismatch, consisting of transplantation from a CMV-positive donor to a CMV-negative recipient, has been associated with a higher risk of PTLD in pre-transplant EBV-seronegative patients, as CMV reactivation is strongly associated with EBV reactivation (34). Moreover, two multivariate models with data from the OPTN/UNOS database revealed that CMV-seronegative kidney recipients were at an elevated risk for PTLD, regardless of donor status (35, 36). By contrast, an analysis of kidney recipients stratified by both EBV and CMV found that CMV status did not make a significant contribution to risk (33), and CMV disease at any stage prior to diagnosis was not associated with an increased risk of PTLD (37). Thus, the relationship between CMV and PTLD remains unclear, and, in this case, the lack of information on CMV-IgM and CMV-IgG status in the donor prevents a definitive determination. Therefore, the pre-transplant CMV serologic status of the donor/recipient is necessary, and close monitoring of transplant recipients who are positive for both EBV-DNA and CMV-DNA after transplantation is suggested.

Pharmacologic immunosuppression compromises the ability of EBV-naive patients to establish an immune response, with high-dose immunosuppressive drug regimens being a risk factor for PTLD (19). Lung transplant recipients have a higher rate of PTLD than recipients of other solid organs, a difference likely due to the greater amount of lymphoid tissue in lung allografts and the increased need for immunosuppression. Moreover, treatment with calcineurin inhibitors, such as tacrolimus and cyclosporine, was found to increase the incidence of PTLD (38). The availability of drug-level monitoring reduced the incidence of PTLD (39). The patient described in the present study received an immunosuppressive regimen consisting of a calcineurin inhibitor and glucocorticoids, with drug level monitoring suggesting that drug concentrations were not abnormally high during his hospitalization. His T-cell counts were below normal for 45 days after lung transplantation, although they tended to increase 60 to 80 days after transplantation; his CD4+/CD8+ ratio was consistently <1.0. Moreover, all of his immune cell counts remained below normal after CT scans showed the shadow of a mass, especially during the later stages of the disease (**Figures 1A–C**).

Other possible risk factors for PTLD after lung transplantation include age, HLA mismatch, and cytokine gene polymorphisms (1, 40). The risk of PTLD decreased with increasing age for subjects younger than 45 years at time of transplant (HR, 0.79 per 5-year increase; 95%CI, 0.7–0.89) (40). The HLA matching status between donor and recipient may affect the development of PTLD. For example, a Swedish study of 553 hematopoietic stem cell transplantation recipients between 1996 and 2004 showed that the incidence of PTLD was 2.5%, with HLA mismatch being a risk factor for PTLD (41). However, the relationship between HLA matching status and PTLD in SOT recipients has been controversial. A study published in 2004 showed that HLA matching status was found to affect the risk of PTLD in EBV-negative recipients receiving EBV-positive organs, with a high degree of HLA matching in EBV-mismatched lung transplant recipients significantly increasing the risk of PTLD (28). On the other hand, some studies revealed that HLA mismatch affected the risk of PTLD; mismatches at the HLA-A1, A24, A26, and B40 loci were associated with an increased risk of PTLD, whereas mismatches at the HLA-B8, DR3, and DR11 loci were associated with a decreased risk of PTLD (40, 42, 43). The present patient had a risk factor for PTLD (56 years of age), but the HLA types in the donor and recipient HLA were not obtained, thus preventing the determination of HLA matching status.

Although the clinical presentation of PTLD can be non-specific, its histopathology is quite distinctive. A biopsy of the lesion is essential. PTLD in the lungs should be differentiated from IgG4-related lung disease, lymphomatoid granulomatosis (44), and infections with bacterial, fungal, and parasitic pathogens. Unfortunately, due to his history of invasive pulmonary aspergillosis, only invasive fungal disease was first considered when lung CT showed multiple bilateral nodules of variable size in this patient, indicating the need for a differential diagnosis and suspicion of PTLD. A prior PET-CT and biopsy are needed to diagnose PTLD.

The definitive test for detecting EBV in a tumor is EBER *in situ* hybridization of paraffin-embedded tissue sections. EBER staining is sensitive and specific for latent EBV infection and allows the virus to be localized to specific cell types by microscopic interpretation (21). The present patient underwent a CT-guided transthoracic lung biopsy, with the biopsy evaluated histopathologically and by NGS. NGS showed the presence of EBV, and tissue biopsy showed PTLD (B cells, monomorphic). NGS technology is an alternative virus nucleic acid detection method. This can obtain the genome sequence of the virus, and by comparing it with a reference genome sequence, mutations, and haplotypes can be identified (45). Liu et al. reported the genome sequencing of the first clinical isolate of EBV obtained from a nasopharyngeal carcinoma using NGS technology (46), and then EBV-associated PTLD was considered in the present patient.

Methods to prevent PTLD and ensure early detection and diagnosis are critical for transplant patients. Because PTLD was associated with EBV in 60–80% of cases (26), monthly monitoring of EBV viral load for 1 year in EBV-naive recipients following solid organ transplantation was recommended by the American Society of Transplantation (47). Moreover, guidelines from the American Society of Transplantation Infectious Diseases Community of Practice suggested that patients who receive seropositive donor organs should be monitored weekly to biweekly during the first posttransplant year until EBV DNAemia is detected. Following the detection of EBV DNAemia, patients should be monitored weekly during the initial acute phase of infection, then less frequently until a “set point” is achieved (weak/very low). Less frequent initial monitoring (monthly) for community-acquired infections should be considered in seronegative patients who receive seronegative donor organs (19). To date, however, there are no specific guidelines on the threshold of EBV-DNA load for therapeutic intervention or the source to be monitored (blood, BALF) (13, 19). Treatment guidelines are needed, as is additional research on EBV-associated PTLD. Monitoring EBV load in at-risk patients is a reasonable strategy to identify impending EBV-positive disease, with a sudden increase in EBV load being sensitive but not highly specific for PTLD (48). In the present case, the application of NGS was only used for pathogenetic examination but not for viral load; we could not draw any conclusions about EBV load from NGS. Quantitative PCR of BALF may be an alternative choice for monitoring EBV load in high-risk PTLD patients (49). Moreover, Sen et al. revealed that miR-194, 17, 19, and 106a are decreased in plasma and EBV+PTLD tumors, showing the potential use of host factors like microRNAs as an indicator of PTLD (50).

The mainstay of PTLD treatment is the reduction of immunosuppression. Many patients may require additional treatments, such as rituximab, chemotherapy, and adoptive immunotherapy, with radiation and surgical therapy reserved for select individuals (18, 19). However, reduction of immunosuppression may theoretically increase the risk of subsequent development of chronic lung allograft dysfunction, which is the major cause of late mortality following lung transplantation (51). The condition of the present patient was too poor to allow any PTLD treatment. Methods are needed to balance immunosuppressive regimens with infection and PTLD.

The antiviral agents ganciclovir and acyclovir have been used for prophylaxis and treatment of EBV-related PTLD. Patients who received antiviral agents during and for up to 3 months after treatment with antilymphocyte agents were shown to be at lower risk for PTLD than patients who did not receive antiviral therapy (52, 53). In contrast, a case-control study (19) comparing 31 lung transplant recipients with and 62 without PTLD showed that prophylactic antiviral therapy (valganciclovir) during the first 3 months did not reduce the incidence of PTLD (54). Guidelines have recommended that antiviral agents not be used as prophylaxis for early PTLD prevention in EBV-mismatched patients (19).

In conclusion, this study describes a lung transplant recipient who developed and eventually died of EBV-associated PTLD. A history of invasive fungal disease and early detection of A. fumigatus indicated the need for antifungal therapy throughout the course of this disease; this information induced clinicians to think of invasive fungal disease and ignored the possibility of PTLD when lung CT showed multiple bilateral nodules of varying size. EBV was consistently positive since postoperative day 15, but antiviral treatment was never taken after the multiple bilateral nodules were shown. These findings suggest the necessity of monitoring EBV-DNA in lung transplant recipients so as to assess their immune status. Moreover, although uncommon, PTLD should be included in the differential diagnosis of transplant recipients, especially EBV-DNA-positive recipients, who present with clinical symptoms that cannot be explained by conventional diagnoses.

## Data availability statement

The raw data supporting the conclusions of this article will be made available by the authors, without undue reservation.

## Ethics statement

The individual’s written informed consent has been obtained for the release of any potentially identifiable images or data contained in this article, and ethical approval has been obtained from the First Affiliated Hospital of Zhejiang University School of Medicine (IIT20230454A).

## Author contributions

JH participated in patient treatment, data collection, and article writing. Y-WY was responsible for data processing, photo editing, and manuscript formatting. D-SH and X-JL participated in NGS-related data processing, processing of pathological sections, and staining. Y-QZ and H-LC participated in the formulation of the patient’s treatment plan. Y-HX and XZ participated in the formulation of the patient’s treatment plan, proposed research ideas, and guided the writing direction. All authors contributed to the article and approved the submitted version.
